# Twenty-Year Clinical Progression of Dysferlinopathy in Patients from Dagestan

**DOI:** 10.3389/fneur.2017.00077

**Published:** 2017-03-08

**Authors:** Zoya R. Umakhanova, Sergei N. Bardakov, Mikhail O. Mavlikeev, Olga N. Chernova, Raisat M. Magomedova, Patimat G. Akhmedova, Ivan A. Yakovlev, Gimat D. Dalgatov, Valerii P. Fedotov, Artur A. Isaev, Roman V. Deev

**Affiliations:** ^1^Dagestan State Medical Academy, Makhachkala, Russia; ^2^S.M. Kirov Military Medical Academy, Saint Petersburg, Russia; ^3^Kazan Federal University, Kazan, Russia; ^4^Human Stem Cells Institute, Moscow, Russia; ^5^Ryazan State Medical University, Ryazan, Russia; ^6^Voronezh Regional Clinical Hospital No. 1, Voronezh, Russia

**Keywords:** dysferlinopathy, LGMD2B, Miyoshi myopathy, dysferlin, muscular dystrophy

## Abstract

To date, over 30 genes with mutations causing limb-girdle muscle dystrophy have been described. Dysferlinopathies are a form of limb-girdle muscle dystrophy type 2B with an incidence ranging from 1:1,300 to 1:200,000 in different populations. In 1996, Dr. S. N. Illarioshkin described a family from the Botlikhsky district of Dagestan, where limb-girdle muscle dystrophy type 2B and Miyoshi myopathy were diagnosed in 12 members from three generations of a large Avar family. In 2000, a previously undescribed mutation in the *DYSF* gene (c.TG573/574AT; p. Val67Asp) was detected in the affected members of this family. Twenty years later, in this work, we re-examine five known and seven newly affected family members previously diagnosed with dysferlinopathy. We observed disease progression in family members who were previously diagnosed and noted obvious clinical polymorphism of the disease. A typical clinical case is provided.

## Introduction

In 1996, a group of doctors from the Research Institute of Neurology (Russian Academy of Medical Science), described an Avar family originating in a Dagestan mountain village with nine members diagnosed with limb-girdle muscle dystrophy type 2B (LGMD2B) and three members diagnosed with Miyoshi myopathy. Subsequently, a mutation in exon 3 c. 573–574 TG>AT of the *DYSF* gene in a homozygous state with a substitution p. Val67Asp was identified in every affected member of the family (1, 2).

The gene *DYSF* consists of 55 exons and has a size of 150,000 bp (3). Dysferlin is an integrated transmembrane protein with expression widely distributed throughout an organism. It is expressed in striated and cardiac muscle, brain, spleen, small intestine, placenta, and monocytes and is expressed in lower amounts in the liver, lungs, kidneys, and pancreas (4). Matsuda et al. determined that dysferlin interacted with calpain-3 and promoted membrane repair (5). The lack of dysferlin or its insufficient activity results in impaired membrane repair in muscle fibers and, consequently, in its destruction and loss followed by increased blood creatine phosphokinase (CPK) levels (6). Thus, insufficient functional dysferlin leads to necrotic changes and fibrosis in muscles that progress to loss of muscle strength in affected individuals (7). The presence of two (2) or even three different (8) clinical phenotypes in patients with the same mutation of *DYSF* remains unexplained.

The purpose of this study was to evaluate disease progression in patients with dysferlinopathy described in 1996 and to study a clinical pattern in affected family members with newly diagnosed dysferlinopathy.

## Patients and Methods

### Patients

**Figure 1:**
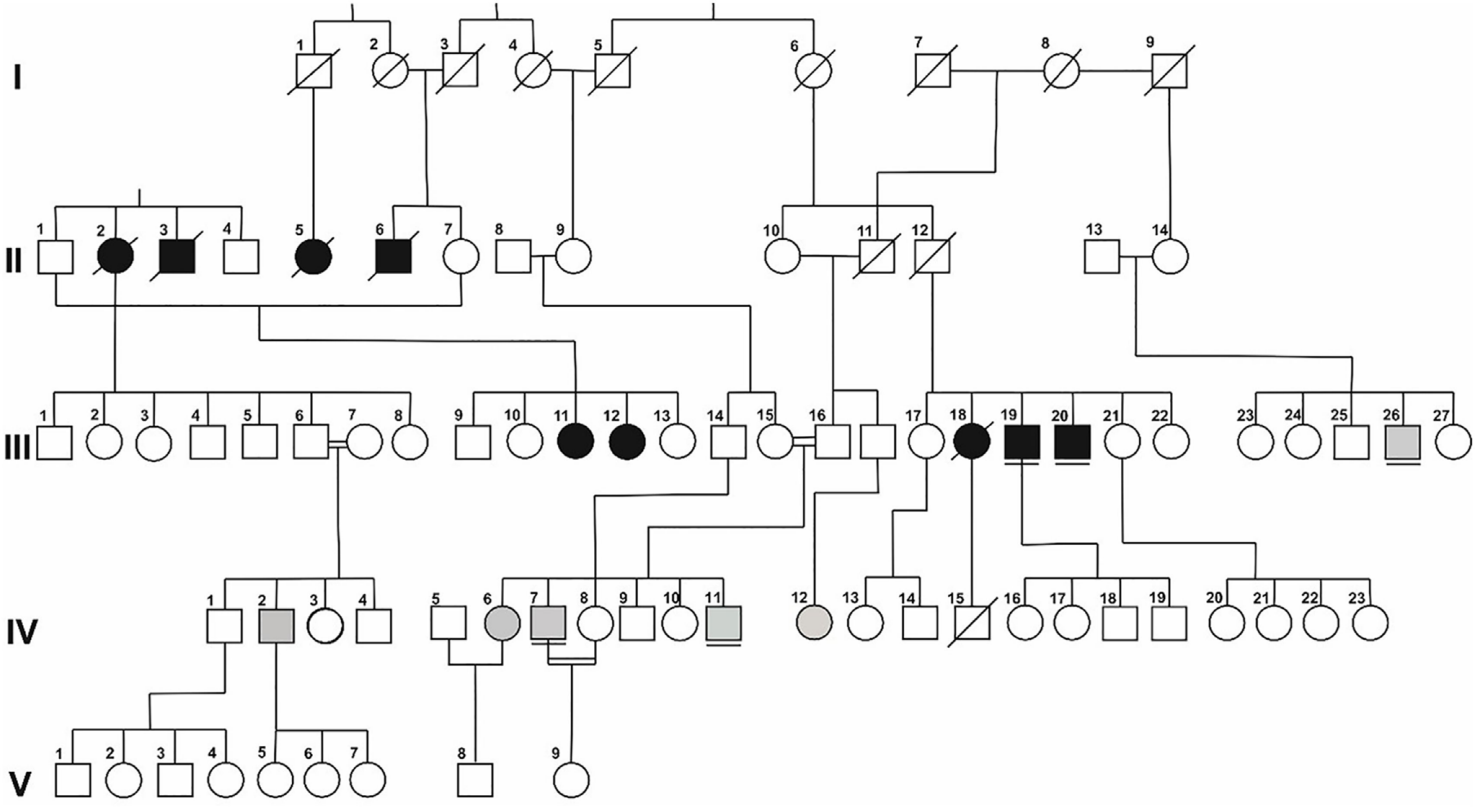
**Simplified pedigree of Family A**. Symbols in black represent patients with a LGMD phenotype; those in gray represent patients with a phenotype of distal myopathy. Underlined symbols highlight previously diagnosed patients.

Twelve patients (nine males and three females) were examined. Five patients originated from the largest branch of the same family (A), which has had manifestations of limb-girdle muscular dystrophy (LGMD) within six generations and were previously described by Illarioshkin et al. (Figure 1). An LGMD phenotype was detected in two patients (A III-19 and A III-20), distal Miyoshi myopathy—in two patients (A III-26 and A IV-7), and asymptomatic increased serum CPK activity levels and electromyographic (EMG) signs of primary muscle disease in one patient (A IV-11). The same mutation in the *DYSF* gene in a homozygous state [TG573/574AT (Val67Asp)] was identified in every patient. All of the patients are close relatives.

All procedures were performed after patients signed a voluntary informed consent form, as required by the Declaration of Helsinki (2013) and the local Ethics Committee (Dagestan State Medical Academy, Makhachkala, Russia).

### Clinical, Genealogical, and Neurological Examination

Five previously diagnosed and seven newly diagnosed patients (A IV-2, A IV-12, B-1, C-1, D-1, E-1, and E-2) underwent clinical and genealogical analyses and a neurological examination (Table 1).

**Table 1:** **Summarized examination results of patients with dysferlinopathy**.

Patient	A III-19	A III-20	B-1	A III-26	A IV-7	A IV-11	A IV-12	A IV-2	C-1	D-1	E-1	E-2
Age at onset, years	16	18	17	21	15	15	28	18	17	19	16	15
Age at examination (1993), years	43	41	4	23	23	6	8	10	8	3	2	5
Age at examination (2013), years	63	61	24	40	42	26	28	30	28	22	22	25
Duration of a disease, years	47	43	7	19	27	11	1	12	11	3	6	10
Phenotype (pattern manifested)	Limb-girdle muscular dystrophy (LGMD)	LGMD	LGMD	Miyoshi	Miyoshi	Miyoshi	Miyoshi	Miyoshi	Prox-distal	Prox-distal	Miyoshi	Miyoshi
Current dominant pattern	Prox-distal UL LL	Prox-distal UL LL	Prox-distal UL LL	Dist.-prox UL LL	Dist.-prox UL LL	Dist.-prox UL LL	Dist.-prox UL	Dist.-prox UL LL	Prox-distal UL LL	Prox-distal UL	Dist.-prox UL LL	Prox-distal UL LL
**TA/G**	**4/5**	**4/5**	**3/3**	**4/3**	**3/3**	**2/3**	**1/2**	**2/2**	**–/1**	**2/2**		**3/2**
**QF/PF**	**5/5**	**5/5**	**3/4**	**3/4**	**3/4**	**1/2**	**1/1**	**–/1**	**–/2**	**1/3**	**1/3**	**1/3**
**MG**	**4**	**4**	**2**	**1**	**1**	**–**	**–**	**–**	**1**	**–**	**2**	**3**
**DA**	**3**	**4**	**1**	**3**	**3**	**2**	**–**	**2**	**2**	**1**	**2**	**2**
**B**	**5**	**4**	**1**	**3**	**2**	**–**	**–**	**2**	**3**	**–**	**1**	**3**
**D + S**	**4**	**4**	**–**	**1**	**–**	**–**	**–**	**–**	**–**	**–**	**–**	**2**
**ES**	**2**	**2**	**–**	**1**	**–**	**–**	**–**	**–**	**–**	**–**	**–**	**–**
Atrophy	TA, G, QF, PF, MG, DA, B, D, S, ES	TA, G, QF, PF, MG, DA, B, D, S, ES	TA, G, QF, PF, DA, B	TA, G, QF, PF, MG, DA, B, D, S	TA, G, QF, PF, DA, B	G, PF, DA	G	TA, G, DA, B	G, PF, DA, B	TA, G, PF, DA	TA, G, QF, PF, MG, DA, B	TA, G, QF, PF, MG, DA, B, D, S
Contractures	AC	AC	–	AC	AC	–	–	–	–	–	AC	AC
No refl.	Abs	Abs	KR, AR	KR, AR	KR, AR	AR	AR	KR, AR	Abs	AR	KR, AR	KR, AR
Motor performance	IC	IC	WG, GM	AbAI	Steppage	AbAI	Normal	AbAI	AbAI, GM	Steppage	Steppage	WG, steppage

*TA, m. tibialis anterior; G, m. gastrocnemius; QF, m. quadriceps femoris; PF, posterior femoral muscles; MG, mm. glutei; DA, distal upper limb muscle; B, m. biceps brachii; D, m. deltoideus; S, muscles of scapula; ER, m. erector spine; Abs, absence of reflexes; AR, Achilles reflex; KR, knee reflex; AC, contracture of Achilles tendon; IC, invalid wheel-chair; AbAI, able to ambulate independently; WG, waddling gait, GM, Gowers’ manoeuvres; UL, upper limbs, LL, lower limbs. In bold frame: muscle power according to MRC scale. The patients previously examined by Illarioshkin et al. (1) are marked in gray*.

### Laboratory and Instrumental Methods

Blood serum CPK activity testing was performed in 12 patients. Nerve conduction study and needle electromyography were performed in three patients (A IV-2, C-1, and D-1). MRI of lower extremity muscles (T1, T2, STIR) was performed in two patients (A IV-2, C-1).

### Histological and Immunohistochemical Analyses

Histological and immunohistochemical analyses were performed for one patient (D-1). A fragment (5 mm^3^) of a lateral portion of the quadriceps femoris muscle was sampled and prepared using the standard procedure; longitudinal and cross-sections of the specimens were stained with hematoxylin and eosin. Immunohistochemistry was carried out with antibodies to dysferlin, dystrophin, alpha-smooth muscle actin, Ki67, CD68, CD4, and CD8.

### Genetic Analysis

A genetic defect was confirmed by PCR with diagnostic primers to a previously known mutation.

### Statistical Analysis

The results of the morphometric analysis are presented as the mean ± SD. Significant changes were assessed by Student’s *t*-test, with *P* < 0.05 as the level of significance.

## Results

### Clinical and Genealogical Analyses

**Figure 2:**
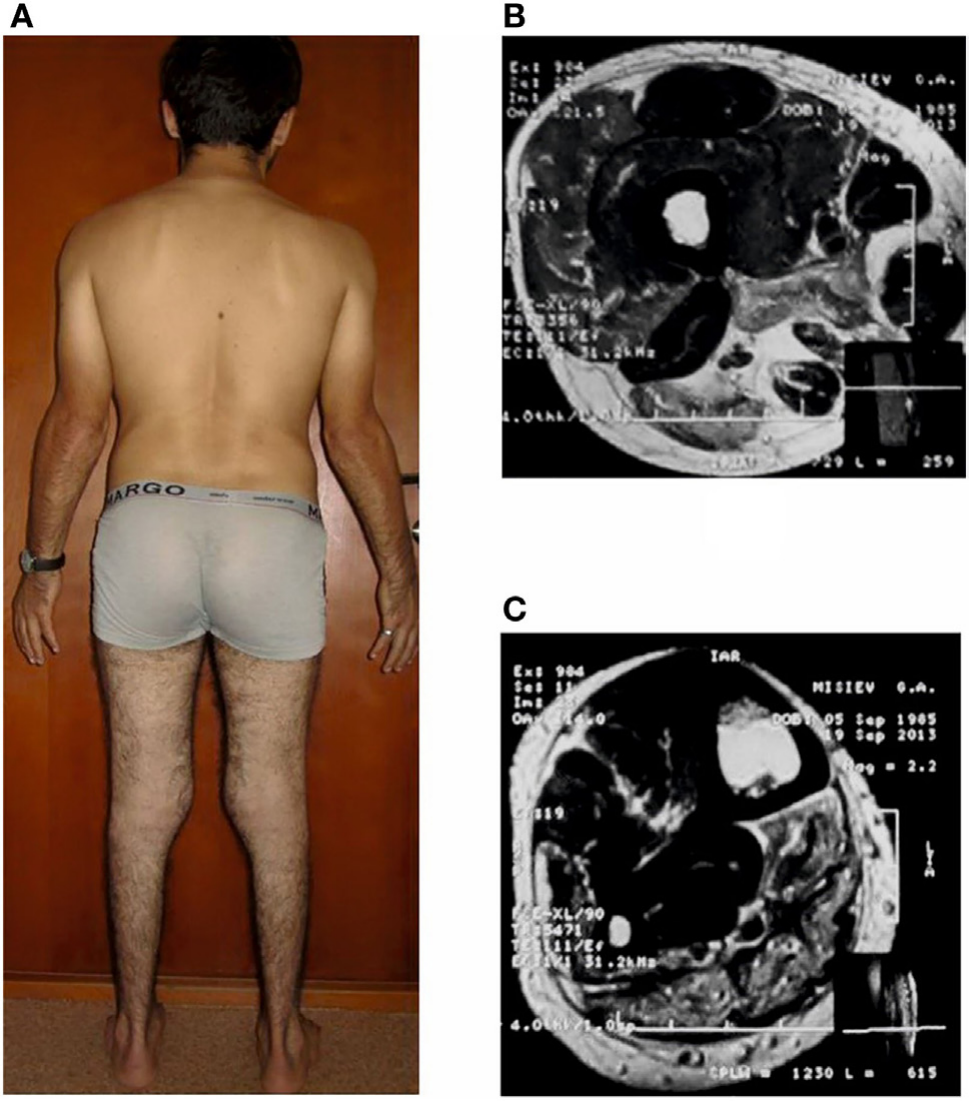
***Patient C-1*, age 28 with distal Miyoshi myopathy (A) and magnetic resonance images (T2-WI) at the level of the middle third of the thigh (B) and lower leg (C)**.

**Figure 3:**
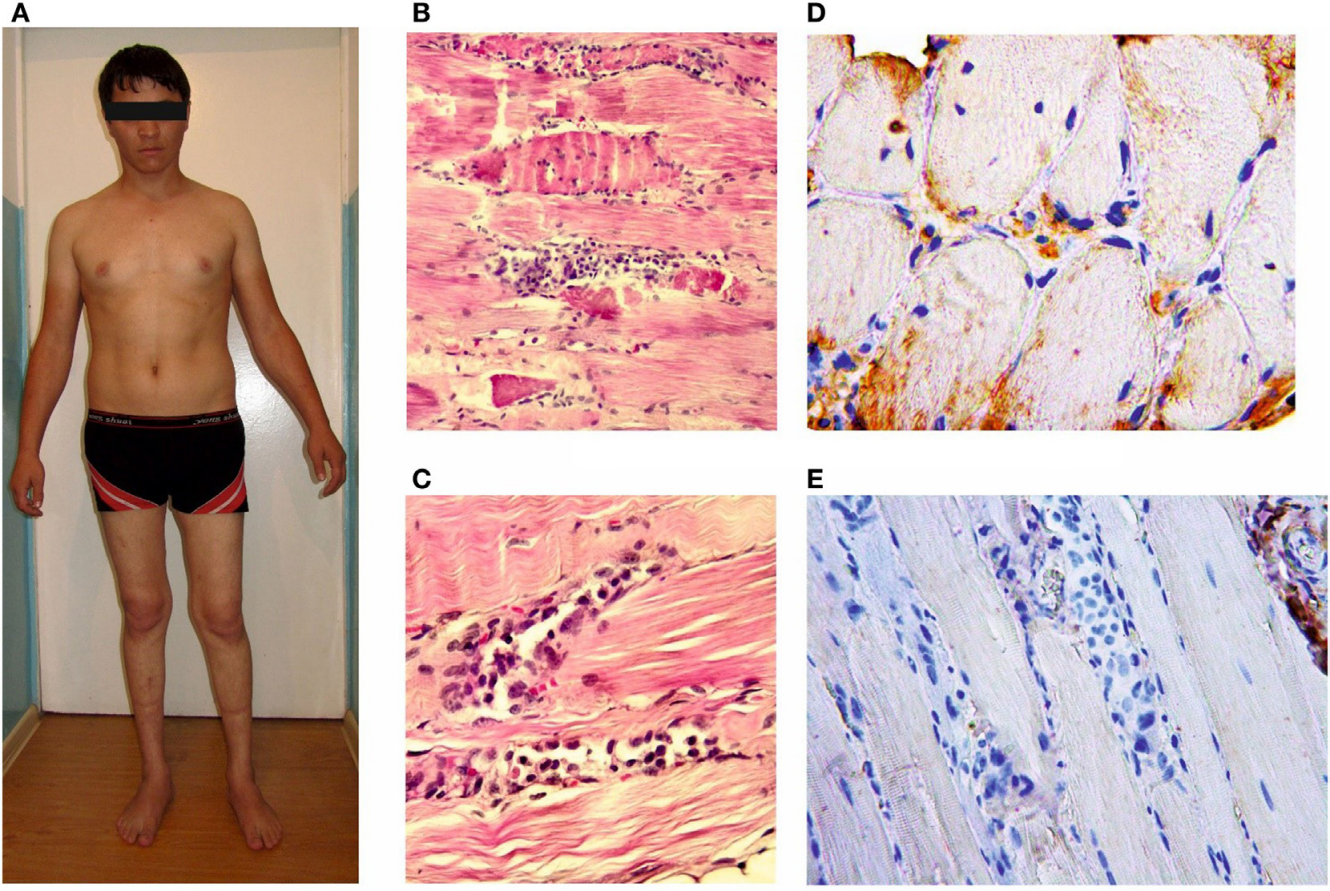
***Patient D-1*, age 22 with the proximal-distal phenotype (A); microslides of m**. vastus lateralis byopsy samples: hematoxylin and eosin staining **(B,C)**; an immunohistochemical reaction with anti-dysferlin antibodies **(D,E)**. The loss of cross-striation, a destruction of some muscle fibers and massive histiocytic infiltration.

Five generations of Family A had 15 members with signs of progressive muscular dystrophy, four intermarriages were identified. Parents and known relatives of five newly diagnosed patients (B-1, C-1 (Figure 2), D-1 (Figure 3), E-1, and E-2) from four other families were clinically healthy. Autosomal recessive inheritance of progressive muscular dystrophy was determined.

### Neurological Examination

A Miyoshi phenotype characterized by primary isolated weakness in distal regions of legs was identified in seven (58%) patients. A phenotype of LGMD characterized by predominant weakness in femoral and pelvic girdle muscles (difficult standing up, waddling gait) was identified in three (25%) patients. Two (16%) patients had proximal–distal phenotype with muscle weakness equally affecting both proximal and distal regions of the lower extremities. Subsequently, muscle weakness and amyotrophy extended upwards.

The mean age of all patients at examination was 34.3 ± 14.4 years (22–62 years). The mean age of symptom onset in all patients occurred during the late teens and was 17.9 ± 3.6 years (15–28 years). All patients had normal physical and mental development within the period preceding disease onset.

### Re-Examined Patients with Previously Diagnosed Dysferlinopathy

Two (A III-19, A III-20) out of five previously examined patients had the LGMD phenotype with normal distal strength due to a proximal myopathy beginning at age 16–18 years. At the age of 41 and 43, both patients presented severe generalized distal weakness with minimal movements in distal regions of the extremities and diffuse atrophy in neck extensors. Upon re-examination at the age of 61 and 63, both patients had severe generalized manifestations of myopathy and were unable to ambulate independently. Both patients had total areflexia and significant contractures in ankle joints, but intact muscles of the face, throat, and sphincters.

Most patients with LGMD (A II-2, A III-3, A III-5, A III-6, A III-11, A III-12) described by Illarioshkin et al. (1) had similar progression, distribution of muscle weakness, and atrophy. They lost their ability to ambulate independently at the age of 40.4 ± 5.4 years (30–45 years).

One patient (A IV-11) was followed up from the age of 6 to 11 without clinical manifestations of myopathy, although he had a threefold increase in the CPK activity level (580 U/L) and intramuscular EMG signs of primary muscle damage (Research Center of Neurology, RAMS). On re-examination at the age of 26, he presented specific symptoms of distal Miyoshi myopathy with paresis and atrophy of the calf muscles. His elder brother (A IV-7) was diagnosed at the age of 23 with the Miyoshi phenotype, which had manifested when he was 16. When re-examining him at the age of 42, we observed marked atrophies and paresis of lower leg muscles and moderate weakness in hamstrings. Atrophy and weakness were more pronounced in the forearm flexors and less evident in the biceps muscles of upper arms, presumably associated with active engagement in freestyle wrestling at the age of 20–25.

The third patient (A III-26) with the Miyoshi phenotype determined at the age of 23 was also examined. The disease manifested at the age of 20, when re-examined at the age of 40, the patient’s severe generalized weakness was worse in the legs. The patient had Achilles tendon contractures and no knee or Achilles reflexes, but was able to walk with support.

### Patients with Newly Diagnosed Dysferlinopathy

The mean age of new patients at examination was 34.3 ± 14.4 years (22–62 years). The mean age of symptom onset was 17.9 ± 3.6 years (15–28 years).

Among the new patients diagnosed, four (A IV-2, A IV-12, E-1, E-2) had a Miyoshi phenotype, two (C-1 and D-1) a proximal–distal phenotype, and one (B-1) a LGMD phenotype. The mean age at disease onset in patients with the Miyoshi phenotype was 19.3 ± 5.9 years (15–28 years), and the mean age at examination was 26.3 ± 3.5 years (22–30 years). One patient (A IV-12) complained at disease onset of weakness in the calves for 1 year that manifested as difficulty standing up on the heels; however, a neurological examination revealed a slightly reduced strength in the posterior muscles of the thighs. All patients with a disease duration ranging from 3 to 10 years (*n* = 4, 66%) had more pronounced distal–proximal myogenic paresis in the lower extremities and mild paresis in the upper extremities with involvement of m. biceps brachii.

One re-examined patient (E-2) with the Miyoshi phenotype had comparatively rapid progression with extension into proximal regions of the upper extremities and upper limb-girdle muscles. Two patients (C-1 and D-1) had a proximal–distal phenotype due to parallel progression of weakness and muscle atrophy of both distal and proximal regions of the lower extremities with subsequent involvement of the upper extremities as in the Miyoshi phenotype.

Subacute myogenic proximal–distal paraparesis of the lower extremities that developed after running (see Case Report) was a hallmark of the dysferlinopathy onset in Patient D-1. All the patients are able to ambulate independently, with steppage observed in 50% of cases due to weakness and atrophy in anterior muscle compartments of the lower leg or diffuse impairment of all of the calf muscles. Knee and Achilles reflexes were absent in all of the patients. Achilles tendon contractures were observed in two patients (E-1, E-2).

One patient (B-1) had the LGMD phenotype, manifested at the age of 17. A neurologic examination of the patient at age 24 found severe generalized weakness, which was worse in legs.

The summarized examination results for the patients are given in Table 1.

### Electromyographic

Two patients (A IV-2, D-1) had nerve conduction study and needle electromyography performed. Normal conduction velocity was recorded along all of the nerves of the arms and legs, with a slightly decreased M-response amplitude. Needle electromyography did not record any spontaneous activity of muscle fibers. Mainly short-duration motor unit action potentials were recorded, with an increased number of polyphasic potentials within the expected range for age in intact muscles (signs of primary muscle disease).

### MRI of Lower Extremity Muscles

Three patients (A IV-2, C-1, and D-1) aged 28, 30, and 23, respectively, with a disease duration of 9.6 ± 3.2 years (6–12 years) received MRI of the lower limb. Decreased muscle volume, diffusely increased intensity of the MR-signal on T1-WI (predominantly) and T2-WI due to the infiltration of degenerated muscle by adipose, and connective tissue were detected. Among the muscles of the lower leg, the earliest changes were typical for caput medialis m. gastrocnemius. The most pronounced changes were observed in m. gastrocnemius, m. soleus, and m. flexor digitorum longus and the least evident changes in the peroneal group and m. tibialis anterior. Among the muscles of the thigh, degenerative changes were more pronounced in caput longum m. biceps femoris and m. adductor magnus (a group of adductors) and less prominent in m. semitendinosus et semimembranosus and m. vastus lateralis.

### Biochemical Analysis

High serum CPK activity level up to 2,000–11,800 U/L (10- to 60-fold to normal) prone to decrease with a patient’s age and disease duration was detected.

### Histological and Immunohistochemical Analyses

Histological and immunohistochemical analyses were performed for only one patient; the results are given in the Section “Case Report.” Immunohistochemical analysis of the patient’s muscle tissue identified of the lack of dysferlin expression in muscle fibers.

### Genetic Analysis

A previously identified mutation in the *DYSF* gene in a homozygous state (c.TG573/574AT; p. Val67Asp) was verified in all the patients.

## Discussion

Three clinical patterns at the stage of disease debut were determined in 12 patients (old and new) from five closely related families originating from isolated mountainous settlements of the Republic of Dagestan: the distal Miyoshi phenotype (seven patients, 58.3%), the proximal–distal phenotype (two patients, 16.6%), and the LGMD2B phenotype (three patients, 25%). Four intermarriages significantly contributed to the incidence of this autosomal recessive disorder.

There is a clear tendency toward the same phenotype within one generation of siblings, indicating the involvement of additional factors modifying expression of the *DYSF* gene. Similar peculiarities were previously reported for Japanese, Canadian, and Italian families with LGMD2B/Miyoshi (9–12) that confirm that there is no correlation between the phenotype and type of mutation. Based on recently published data, the presence of modifying genes (*ANXA2* and *ANXA1*) affecting the outcome can be one possible explanation for this discrepancy.

Heterogeneity is specific for the age of manifestation and, therefore, for the period of asymptomatic disease. The mean age of disease onset for patients of every phenotype was 17.9 ± 3.6 years, with the earliest manifestation occurring at the age of 15 as difficulty walking on the heels. Previous articles reported the debut of dysferlinopathy at the age of 47, as well as an asymptomatic disease until the age of 58, accompanied only by increased serum CPK activity levels (13, 14). Patient A IV-11, with an asymptomatic increased serum CPK activity level and EMG signs of primary muscle disease, had disease onset under the Miyoshi phenotype at age 15. This case confirms the presence of a latent course of dysferlinopathy and supports the possible use of EMG and CPK measurements for screening suspected homozygous carriers of mutations in the *DYSF* gene prior to clinical manifestation.

### Case Report

A male patient at the age of 22 (D-1) with proximal–distal LGMD2B and a disease duration of 3 years is an example of a common diagnostic mistake associated with an acute or subacute onset of muscle weakness after exercise occasionally accompanied with pain and swollen legs. While in the army at the age of 19, the patient had subacute proximal paraparesis of the lower extremities; therefore, biopsy of m. vastus lateralis was performed. In addition to necrotized muscle fibers, lymphocytic infiltration of the endomysium was revealed and was interpreted as a manifestation of acute polymyositis, and corticosteroids were prescribed. The patient took them for 1½ year without any significant therapeutic effect. Knee and Achilles reflexes were absent. There were slight contractures of the Achilles tendons. A lack of dysferlin expression in muscle fibers was detected upon histological re-examination with immunohistochemistry. Corticosteroids were discontinued.

A genetic analysis identified the same missense mutation in exon 3 c. 573–574 TG>AT of the gene *DYSF* in a homozygous state with a substitution p. Val67Asp. This mutation is not found in electronic databases and has not been described in other studies of dysferlinopathy to enable an assumption of a common ancestor for members of these families.

## Concluding Remarks

In this work, an identical mutation confirming the fact of a common ancestor for patients from different families (A, B, C, D, E) with four intermarriages was identified. Patients with dysferlinopathy, both previously described and newly diagnosed were studied. This mutation, located in the *DYSF* gene, presented as three distinct phenotypes: distal Miyoshi, LGMD2B, and proximal–distal phenotype. Patients with a distal phenotype prevailed among those examined. Moreover, we also analyzed the dynamics of progression, sequence of involvement and intensity of the muscular dystrophic process in different groups of muscles over the past 20 years. In two cases (patients A IV-7 and D-1), disease onset was associated with physical activity. Therefore, we suggest that physical exercise is an accelerating and enhancing factor for progression of a muscle dystrophy due to the direct role of dysferlin deficiency in repair disruption.

Similar studies will facilitate the development of distinct diagnostic criteria and provide guidance in optimal patient management by refining the selection of molecular and genetic methods used to verify a diagnosis and produce genetic prognoses for families.

## Author Contributions

ZU and SB: patient examination, medical data collection, and article preparation; SB: electroneuromyography performance; MM: histological and immunohistochemical analysis and article preparation and translation; OC: histological and immunohistochemical analysis; RM and PA: patient examination, MRI performance, and medical data collection; IY: medical data analysis, article preparation, and correction and translation; GD: genetic analysis and genetic consultation; VF: patient examination; AI: patient examination, medical data collection, and scientific consultation; RD: histological and immunohistochemical analysis, medical and scientific consultation, and article preparation, and the concept of this research.

## Conflict of Interest Statement

The authors declare that the research was conducted in the absence of any commercial or financial relationships that could be construed as a potential conflict of interest.

## Abbreviations

bp, base pairs; CPK, creatine phosphokinase; ENMG, electroneuromyography; LGMD, limb-girdle muscular dystrophy; MUAP, motor unit action potentials.

## References

[1] Illarioshkin SN Ivanova-Smolenskaya IA Tanaka H Vereshchagin NV Markova ED Poleshchuk VV Clinical and molecular analysis of a large family with three distinct phenotypes of progressive muscular dystrophy. Brain (1996) 119:1895–909. 10.1093/brain/119.6.1895 9009996

[2] Illarioshkin SN Ivanova-Smolenskaya IA Greenberg CR Nylen E Sukhorukov VS Poleshchuk VV Identical dysferlin mutation in limb-girdle muscular dystrophy type 2B and distal myopathy. Neurology (2000) 55:1931–3. 10.1212/WNL.55.12.1931 11134403

[3] Anderson LVB Davison K Moss JA Young C Cullen MJ Walsh J Dysferlin is a plasma membrane protein and is expressed early in human development. Hum Mol Genet (1999) 8:855–61. 10.1093/hmg/8.5.855 10196375

[4] Aoki M Liu J Richard I Bashir R Britton S Keers SM Genomic organization of the dysferlin gene and novel mutations in Miyoshi myopathy. Neurology (2001) 57(2):271–8. 10.1212/WNL.57.2.271 11468312

[5] Matsuda C Hayashi YK Ogawa M Aoki M Murayama K Nishino I The sarcolemmal proteins dysferlin and caveolin-3 interact in skeletal muscle. Hum Mol Genet (2001) 10(17):1761–6. 10.1093/hmg/10.17.1761 11532985

[6] Liu J Aoki M Illa I Wu C Fardeau M Angelini C Dysferlin, a novel skeletal muscle gene, is mutated in Miyoshi myopathy and limb girdle muscular dystrophy. Nat Genet (1998) 20:31–6. 10.1038/1682 9731526

[7] Bushby KM. Dysferlin and muscular dystrophy. Acta Neurol Belg (2000) 100:142–5. 11098285

[8] Vilchez JJ Gallano P Gallardo E Lasa A Rojas-Garcia R Freixas A Identification of a novel founder mutation in the DYSF gene causing clinical variability in the Spanish population. Arch Neurol (2005) 62:1256–9. 10.1001/archneur.62.8.1256 16087766

[9] Weiler T Bashir R Anderson LVB Davison K Moss JA Britton S Identical mutation in patients with limb girdle muscular dystrophy type 2B or Miyoshi myopathy suggests a role for modifier gene(s). Hum Mol Genet (1999) 8:871–7. 10.1093/hmg/8.5.871 10196377

[10] Nakagawa M Matsuzaki T Suehara M Kanzato N Takashima H Higuchi I Phenotypic variation in a large Japanese family with Miyoshi myopathy with nonsense mutation in exon 19 of dysferlin gene. J Neurol Sci (2001) 184:15–9. 10.1016/S0022-510X(00)00484-6 11231027

[11] Kawabe K Goto K Nishino I Angelini C Hayashi YK. Dysferlin mutation analysis in a group of Italian patients with limb-girdle muscular dystrophy and Miyoshi myopathy. Eur J Neurol (2004) 11:657–61. 10.1111/j.1468-1331.2004.00755.x 15469449

[12] Harris E Bladen CL Mayhew A James M Bettinson K Moore U The Clinical Outcome Study for dysferlinopathy: an international multicenter study. Neurol Genet (2016) 2(4):e89. 10.1212/NXG.0000000000000089 27602406 PMC4994875

[13] Suzuki N Aoki M Takahashi T Takano D Asano M Shiga Y Novel dysferlin mutations and characteristic muscle atrophy in late-onset Miyoshi myopathy. Muscle Nerve (2004) 29(5):721–3. 10.1002/mus.20025 15116377

[14] Nguyen K Bassez G Krahn M Bernard R Laforêt P Labelle V Phenotypic study in 40 patients with dysferlin gene mutations: high frequency of atypical phenotypes. Arch Neurol (2007) 64:1176–82. 10.1001/archneur.64.8.1176 17698709

