# Corrigendum: Twenty-Year Clinical Progression of Dysferlinopathy in Patients from Dagestan

**DOI:** 10.3389/fneur.2017.00145

**Published:** 2017-04-18

**Authors:** Zoya R. Umakhanova, Sergey N. Bardakov, Mikhail O. Mavlikeev, Olga N. Chernova, Raisat M. Magomedova, Patimat G. Akhmedova, Ivan A. Yakovlev, Gimat D. Dalgatov, Valerii P. Fedotov, Artur A. Isaev, Roman V. Deev

**Affiliations:** ^1^Dagestan State Medical Academy, Makhachkala, Russian Federation; ^2^S.M. Kirov Military Medical Academy, Saint Petersburg, Russian Federation; ^3^Kazan Federal University, Kazan, Russian Federation; ^4^Human Stem Cells Institute, Moscow, Russian Federation; ^5^Voronezh Regional Clinical Hospital No.1, Voronezh, Russian Federation; ^6^Ryazan State Medical University, Ryazan, Russian Federation

**Keywords:** dysferlinopathy, LGMD2B, Miyoshi myopathy, dysferlin, muscular dystrophy

**The “Funding” section should be:**

This work was funded by Human Stem Cells Institute PJSC and Roman V. Deev. Theoretical part of this work was supported by Russian Scientific Foundation grant (14-15-00916). Ivan A. Yakovlev and Mikhail O. Mavlikeev were supported by the Russian Government Program of Competitive Growth of Kazan Federal University.

The authors apologize for this error and state that this does not change the scientific conclusions of the article in any way.

## Conflict of Interest Statement

The authors declare that the research was conducted in the absence of any commercial or financial relationships that could be construed as a potential conflict of interest.

